# Publisher’s note

**DOI:** 10.1152/function.023.2026_NOT

**Published:** 2026-05-28

The American Physiological Society (APS) has been made aware that the required Editor disclosure statement was not included in several articles coauthored by Editors of *Function*. APS takes responsibility for this omission. The absence of the disclosure resulted from a metadata transfer error during the submission to production workflow, which prevented the statement from appearing in the final version of record.

The articles affected are listed below, with the relevant Editor-Authors designated by italics:

**Schenk S, Sagendorf TJ, Many GM, Lira AK, de Sousa LGO, Bae D, Cicha M, Kramer KS, Muehlbauer M, Hevener AL, Rector RS, Thyfault JP, Williams JP, Goodyear LJ, Esser KA, Newgard CB, *Bodine SC*; The MoTrPAC Study Group.** Physiological adaptations to progressive endurance exercise training in adult and aged rats: insights from the Molecular Transducers of Physical Activity Consortium (MoTrPAC). *Function* 5: zqae014, 2024. First published March 28, 2024. doi:10.1093/function/zqae014.

**Kedia S, Awal NM, Seddon J, *Marder E*.** Sulfonylurea receptor pharmacology alters the performance of two central pattern generating circuits in *Cancer borealis*. *Function* 5: zqae043, 2024. First published September 18, 2024. doi:10.1093/function/zqae043.

After publication of these articles, APS confirmed that the necessary blinding was in place during peer review to exclude the Editor from accessing any information regarding peer review of their article.

The updated disclosure statement is as follows:

## DISCLOSURES

[*Authors*] are editors of *Function* and were not involved and did not have access to information regarding the peer-review process or final disposition of this article. An alternate editor oversaw the peer-review and decision-making process for this article.

